# Giant negative magnetoresistance induced by the chiral anomaly in individual Cd_3_As_2_ nanowires

**DOI:** 10.1038/ncomms10137

**Published:** 2015-12-17

**Authors:** Cai-Zhen Li, Li-Xian Wang, Haiwen Liu, Jian Wang, Zhi-Min Liao, Da-Peng Yu

**Affiliations:** 1 State Key Laboratory for Mesoscopic Physics, Department of Physics, Peking University, Beijing 100871, China; 2 International Center for Quantum Materials, School of Physics, Peking University, Beijing 100871, China; 3 Collaborative Innovation Center of Quantum Matter, Beijing, China

## Abstract

Dirac electronic materials beyond graphene and topological insulators have recently attracted considerable attention. Cd_3_As_2_ is a Dirac semimetal with linear dispersion along all three momentum directions and can be viewed as a three-dimensional analogue of graphene. By breaking of either time-reversal symmetry or spatial inversion symmetry, the Dirac semimetal is believed to transform into a Weyl semimetal with an exotic chiral anomaly effect, however the experimental evidence of the chiral anomaly is still missing in Cd_3_As_2_. Here we show a large negative magnetoresistance with magnitude of −63% at 60 K and −11% at 300 K in individual Cd_3_As_2_ nanowires. The negative magnetoresistance can be modulated by gate voltage and temperature through tuning the density of chiral states at the Fermi level and the inter-valley scatterings between Weyl nodes. The results give evidence of the chiral anomaly effect and are valuable for understanding the Weyl fermions in Dirac semimetals.

## Abstract (web summary)

Dirac semimetals possess an electronic dispersion relation which is linear in three dimensions, making them three-dimensional analogues of graphene. Here, the authors report large negative magnetoresistance in single-crystal Cd_3_As_2_ nanowires, evidencing a sought-after chiral anomaly effect.

## Graphical abstract

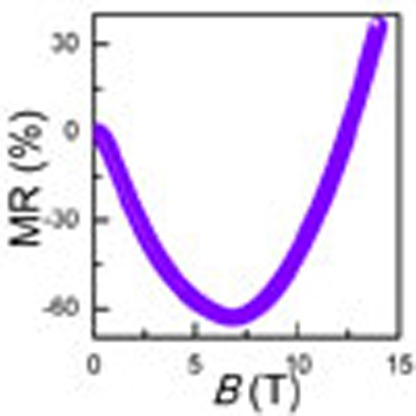

Dirac materials such as graphene and topological insulators have attracted great attention recently, possessing relativistic and massless Dirac fermions with linear energy dispersion in two-dimensional momentum space^1^,^2^,^3^. In extending to three-dimensional (3D) momentum space, the linear energy bands along all three directions cross at crystal symmetry-protected Dirac points, which has been found in topological Dirac semimetals^4^,^5^. Recently, it is predicted that A_3_Bi (refs 6, 7, 8, 9); (A=Na, K, Rb) and Cd_3_As_2_ (refs 10, 11, 12, 13, 14, 15, 16, 17, 18) are candidates for 3D topological Dirac semimetal materials in theory. Thereafter, the unique energy band structures have been identified by angle-resolved photoemission spectroscopy^11^,^12^,^13^ and scanning tunnelling microscope^18^ experiments in Cd_3_As_2_. Moreover, transport measurements^14^,^15^,^16^,^17^ of Cd_3_As_2_ bulk crystals exhibit giant positive magnetoresistance (MR), non-trivial quantum oscillations and Landau level splitting under magnetic field, which confirm the existence of a 3D Dirac semimetal phase in Cd_3_As_2_. The Dirac point in a 3D Dirac semimetal is composed of two overlapping Weyl nodes with opposite chirality, which can be separated in momentum space by breaking time-reversal symmetry or spatial inversion symmetry^10^. Applying a magnetic field can break up the time-reversal symmetry, resulting in the transformation of Dirac semimetals into Weyl semimetals. The separated Weyl nodes are distributed along the external magnetic field and the distance between the two Weyl nodes is proportional to the magnitude of the magnetic field^19^. An important feature of Weyl semimetals is the chiral anomaly effect^20^,^21^,^22^,^23^, where the charges at the two Weyl nodes are with opposite chirality. In the presence of a magnetic field and an electric field which are parallel with each other, the Weyl fermions residing at one Weyl node transport to the other one with opposite chirality, resulting in non-conserved chiral charges and negative MR. Nevertheless, the negative MR has yet to be observed in Cd_3_As_2_, a widely investigated Dirac semimetal system, which would evidence the long anticipated chiral anomaly effect.

To observe the chiral anomaly induced negative MR, it is required that the carrier density is low enough that the Fermi level is located close to the Dirac point, that is, *k*_F_<*k*_D_, where *k*_F_ is the Fermi wave vector and *k*_D_ is the location of the Dirac point referring to the high symmetric Γ point^19^,^21^. Previous experiments on Cd_3_As_2_ bulk materials indicated that the Fermi level is ∼200 meV above the Dirac point^18^ and the carrier density is in the range of 4.4 × 10^18^−1.5 × 10^19^ cm^−3^ (ref. 14), which hinders the observation of the chiral anomaly effect. Here we employ single crystal Cd_3_As_2_ nanowires with low carrier concentration to study the chiral anomaly effect on the transport properties. As magnetic field is parallel to electric field, a notable negative MR is observed with magnitude of −63% at 60 K and −11% at 300 K, giving a clear evidence for chiral anomaly.

## Results

### Microstructures

**Figure 1:**
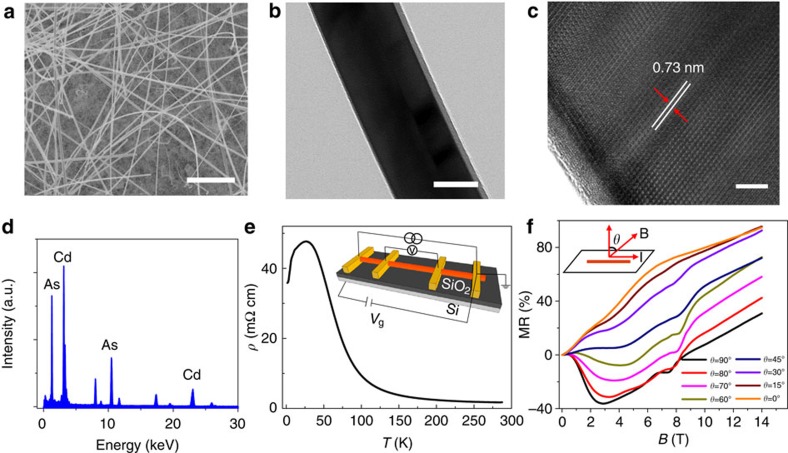
Characterization of synthesized Cd_3_As_2_ nanowires. (**a**) SEM image of the nanowires. Scale bar, 10 μm. (**b**) TEM image of a nanowire with diameter ∼100 nm. Scale bar, 50 nm. (**c**) High-resolution TEM image of a nanowire. The 0.73 nm interplanar spacing indicates the [112] growth direction. Scale bar, 5 nm. (**d**) The energy-dispersive X-ray spectroscopy spectrum of the nanowire. The atomic ratio of Cd and As is approximately 3:2. (**e**) Temperature dependence of resistivity of a nanowire device with diameter ∼200 nm (Sample 1), showing semiconducting-like behaviour. The resistivity reaches maximum at around 26 K. Inset: schematic of the nanowire device with four-probe measurement configuration. The Si substrate is used as the back gate electrode. (**f**) MR of the nanowire device measured at 1.5 K whilst varying the magnetic field from *B*⊥*E* (*θ*=0°) to *B*//*E* (*θ*=90°). Inset: schematic of the relative orientations of *B* and *E*. The applied constant current is along the nanowire [112] direction, and the magnetic field is applied in the plane defined by the nanowire direction and the vector normal to the substrate. *θ* is an angle between *B* and normal direction of the substrate.

Cd_3_As_2_ is a group II–V compound and belongs to body-centred tetragonal crystal system with *I*4_1_/*acd* space group^24^. The Cd_3_As_2_ nanowires were synthesized via chemical vapour deposition method. The nanowires have a large aspect ratio and demonstrate great flexibility, as shown by the scanning electron microscope (SEM) image in Fig. 1a. The nanowires possess a length up to several hundred microns and the diameter ranges from 50 to 500 nm. Figure 1b shows a transmission electron microscope (TEM) image of a typical nanowire with diameter of ∼100 nm. The high-resolution TEM image shown in Fig. 1c demonstrates the single crystal nature of the nanowire. The 0.73 nm interplanar spacing suggests the [112] growth direction of the nanowires. The energy-dispersive X-ray spectroscopy acquired from nanowire in the TEM is shown in Fig. 1d, and further quasi-quantitative analysis indicates that the atomic ratio of Cd and As is consistent with the stoichiometric composition of Cd_3_As_2_.

### Carrier density

Individual Cd_3_As_2_ nanowires were fabricated to contact with Au electrodes on a Si substrate with a 285 nm SiO_2_ layer. The Si substrate serves as the back gate. The schematic diagram of the field-effect transistor with four-probe measurement configuration is shown in the inset in Fig. 1e. The transport properties of a typical Cd_3_As_2_ nanowire device with diameter ∼200 nm (Sample 1) were measured (Supplementary Fig. 1). The temperature dependence of resistivity shown in Fig. 1e demonstrates that the resistivity increases as the temperature decreases from 300 to 26 K. While below 26 K, the resistivity decreases with decreasing temperature. As far as we know, it is very different from the metallic behaviour of Cd_3_As_2_ bulk crystal^14^. The Cd_3_As_2_ bulk materials usually have high carrier density and the Fermi level is far above Dirac points^14^,^18^. Distinctively, the as-grown Cd_3_As_2_ nanowires show a very low carrier density (Supplementary Fig. 2), which renders the Fermi level very close to the Dirac point. The calculated carrier density ∼10^17^ cm^−3^ in the nanowire is much smaller than that in bulk Cd_3_As_2_. The angle-resolved photoemission spectroscopy measurement shows that the Dirac point in bulk Cd_3_As_2_ is located at about −200 meV, corresponding to a carrier concentration ∼5.2 × 10^18^ cm^−3^ (ref. 12). The carrier density of 3D Dirac semimetal can be estimated according to 
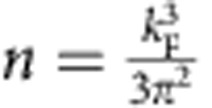
 (ref. 14). Because of the linear dispersion relation of *E*_F_∝*k*_F_, the Fermi energy can be obtained by *E*_F_∝*n*^1/3^. Therefore, the *E*_F_ is estimated to be ∼54 meV in our nanowires.

### Non-metallic *ρ*–*T* behaviours

Similar semiconducting-like *ρ*–*T* behaviours have been found in other Dirac semimetal bulk materials, such as ZrTe_5_ (ref. 25) and Na_3_Bi (ref. 26), which were ascribed to the low carrier density in those materials. Due to the gapless energy band of the semimetal, the holes in the valence band can be thermally activated as the Fermi level is close to the Dirac point. As temperature decreases, the reduced thermal activation is responsible for the semiconducting-like *ρ*–*T* behaviour. After reaching a critical low temperature, the thermal energy is low enough and cannot activate the electrons to the conduction band above the Fermi level, resulting in the invalid of the thermal activation mechanism and a metallic-like *ρ*–*T* behaviour at low temperatures. Compared with Cd_3_As_2_ bulk crystals, one advantage of the nanowires is that it has high crystal quality and can avoid the carrier doping from the vacancies in the crystal. By employing the nanowire field-effect transistor devices, the *ρ*–*T* dependence can be changed from semiconducting-like to metallic-like behaviour as the Fermi level is tuned away from the Dirac point (Supplementary Fig. 3).

### Angular dependence of MR

The angular dependence of MR was performed by varying the relative orientation between the current direction and the magnetic field direction in the plane perpendicular to the substrate. As shown in the inset in Fig. 1f, *θ* represents the angle between the magnetic field direction and normal direction of the substrate. At 1.5 K, the MR defined as 
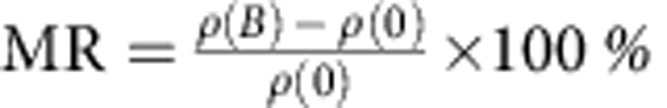
 at different *θ* are presented in Fig. 1f. For *B*⊥*E* (*θ*=0°), there is a positive MR. The positive MR under perpendicular magnetic field can reach up to 2,140% at intermediate temperature (Supplementary Fig. 4). The MR decreases apparently as the direction of the magnetic field changes from *B*⊥*E* (*θ*=0°) to *B*//*E* (*θ*=90°), as shown in Fig. 1f. It is interesting to note that there is a negative MR as *B*//*E*. To verify the experimental results, we have measured >10 samples. The results of negative MR are repeatable in the nanowires with different diameters (up to ∼450 nm, Supplementary Fig. 5) and in the nanoplate with thickness ∼540 nm (Supplementary Fig. 6).

### Negative MR under *B*//*E*

**Figure 2:**
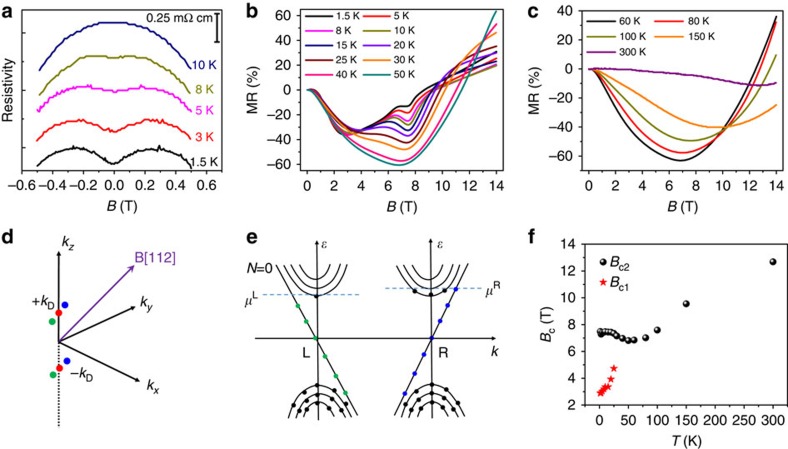
Negative MR under *B*//*E* of a nanowire with diameter ∼200 nm. (**a**) Magnetic field dependence of the resistivity under *B*<0.5 T for sample 1, showing the weak anti-localization effect at low temperatures. (**b**,**c**) MR measured at temperatures from 1.5 to 300 K. The negative MR is observed at all temperatures. The amplitude of the negative MR increases as the temperature increases and reaches a maximum at ∼60 K, then decreases with further increasing temperature. (**d**) Illustration of split of Dirac points into Weyl nodes under an external magnetic field in the [112] direction. The Dirac points at ±*k*_D_ in the Brillouin zone of Cd_3_As_2_ are marked by red dots. The green (blue) dots correspond to the separated left-handed (right handed) Weyl nodes along the direction of magnetic field. (**e**) Schematic of the chiral anomaly in Weyl semimetal. L and R represent the left-handed and right-handed chiralities in the lowest Landau levels (*N*=0), respectively. A magnetic field parallel to the electric field generates an imbalance of chiral charges between the two opposite Weyl nodes. (**f**) The critical magnetic field *B*_C_ as a function of temperature. The *B*_C_ corresponds to the minimum point of each MR curve presented in **b**,**c**.

Typical results of the negative MR from the nanowire device with diameter ∼ 200 nm (Sample 1) are presented in Fig. 2. The low field behaviours of the MR under *B*//*E* and at different temperatures are shown in Fig. 2a. At zero *B*, an obvious dip of the MR is exhibited at 1.5 K, and then gradually disappears with increasing temperature to 10 K. This MR dip is ascribed to the weak anti-localization effect as a result of strong spin–orbit interactions in Cd_3_As_2_. Nevertheless, the negative MR under low magnetic field is very robust against temperature. To study the negative MR in more details, the MR curves at various temperatures under *B*//*E* are given in Fig. 2b,c. The maximum magnitude of the negative MR is up to −63% at 60 K and 7 T. A negative MR ∼−11% is still observed at 300 K. Because the negative MR is rather robust and survives at room temperature, the weak localization effect due to the quantum interference is not the origin of the observed negative MR. Recently, negative MR has been observed in Dirac semimetal ZrTe_5_ (ref. 25), Na_3_Bi (ref. 26) and Bi_1−*x*_Sb_*x*_ (ref. 27) and Weyl semimetal TaAs (refs 28, 29) systems, which was ascribed to the chiral anomaly induced charge pumping effect. Cd_3_As_2_, as 3D Dirac semimetal, its Dirac point described by four-component Dirac equations is composed by two Weyl nodes with opposite chirality (right handed or left handed). Applying a magnetic field, these two Weyl nodes would be separated in momentum space along with the direction of the magnetic field^19^,^27^. For the Cd_3_As_2_ system, there are a pair of Dirac points along *k*_z_ direction and located at ±*k*_D_ near the high symmetric point Γ, as marked by red dots in Fig. 2d. The magnetic field applied in [112] direction splits the Dirac points into Weyl nodes along the direction of magnetic field, as marked by the green and blue dots in Fig. 2d, leading to the formation of Weyl fermions. Initially the right- and left-handed fermions in the different Weyl nodes have equal chemical potential *μ*^R^=*μ*^L^. In the presence of parallel electric field, there would be an imbalance (*μ*^R^≠*μ*^L^) between two Weyl nodes with opposite chirality, which induces a charge pumping from one Weyl node to another with opposite chirality, corresponding to the chiral anomaly as illustrated in Fig. 2e. In such a case, the continuity equation of right- or left-handed Weyl node takes the form of 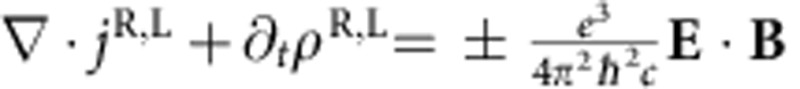 (ref. 30). The chiral charge at a single Weyl node is not conserved, which is the so-called chiral anomaly. The charge depleted at one Weyl node will be generated at the other node with opposite chirality (charge pumping) and thus the charge is conserved over all the system. There is a net current generation as a result of chiral imbalance in the form of 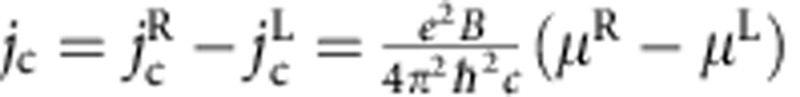, where *j*_c_ is named as chiral current. Since the chiral current is in the direction of electric field, a negative MR will be induced.

It is worth to note that the MR curves at low temperatures have two minima at the critical magnetic field *B*_C1_ and *B*_C2_, as shown in Fig. 2b,c and summarized in Fig. 2f. The first minimum point at about 3 T becomes indistinct as temperature increases to 30 K. The low carrier density of our samples allows the electrons entering into the lowest Landau level at a relatively weak field ∼2–3 T, and there is an inflection in the MR curve. Notably with increasing temperatures, the increase of carrier density due to thermal activation results in an increase in *B*_C1_. Due to the combined Zeeman and orbital terms, it was theoretically predicted that the lowest Landau level (*N*=0) in the conduction band splits into two sublevels by applying a magnetic field^10^,^18^. At low temperature of 1.5 K, the upturned MR above ∼3 T is due to the splitting of the lowest Landau level. As temperature increases to ∼30 K, the thermal broadening of the two sublevels becomes responsible for the vanishing of the upturned MR at low magnetic field (Fig. 2b). The second minimum point at about 7.5 T seems to be more robust and becomes dominant for temperatures higher than 20 K. Recent theory predicts that applying a large magnetic field deviating from [001] direction will induce an energy gap due to breaking of *C*4 crystal symmetry^18^. As the Fermi level lowers down and gets closer to the field-induced gap, the break of the chiral anomaly mechanism results in the attenuation of the negative MR. The critical magnetic field *B*_C2_ has a weak temperature dependence below 80 K and then shifts to high magnetic field at higher temperature (Fig. 2c), in agreement with the Zeeman splitting Δ*E*_z_(*B*=7.5 T)=*gμ*_B_B∼81 K considering the *g* factor ∼ 16 for Cd_3_As_2_ (ref. 31). As temperature is further increased >80 K, the thermal activation of carriers can overcome the magnetic field-induced energy gap, leading to the increase of *B*_C2_. Similar to the *R*–*T* behaviour, the negative MR is also slightly different in details from sample to sample, which is due to the different residual carrier density in different samples. For the sample retaining non-metallic *R*–*T* behaviour at low temperatures, the magnitude of the negative at 1.5 K is small (Supplementary Fig. 7). The different carrier density of different samples leads to the deviation in MR behaviour, such as the different magnitude of negative MR and the critical magnetic field.

At room temperature, the negative MR dominates the entire range from 0 to 14 T (Fig. 2c). Although, there is a bandgap near the Dirac point under high magnetic field, the Weyl semimetal phase and chiral states can be maintained in the linear bands away from the Dirac point as long as the induced gap is small^6^,^19^. The maintained negative MR at room temperature indicates that the chiral states are robust against thermal perturbation. From Hall measurements of a Cd_3_As_2_ nanoplate (Supplementary Fig. 8), the carrier density at 300 K is estimated to be 5.31 × 10^17^ cm^−3^. With such a low value, the corresponding Fermi wave vector is about *k*_F_=0.025 Å^−1^ and the Fermi level *E*_F_=*ħυ*_F_*k*_F_ can be estimated to be 82 meV assuming an applicable Fermi velocity *υ*_F_=5 × 10^5^ ms^−1^, which satisfies the condition of *k*_F_<*k*_D_ for the observation of the chiral anomaly effect in Dirac semimetals. With increasing temperature, thermal activation and thermal-related scatterings will gradually obscure the negative MR, which is responsible for the observation that the magnitude of the negative MR at 300 K is much smaller than that at low temperatures.

### Gate modulation

**Figure 3:**
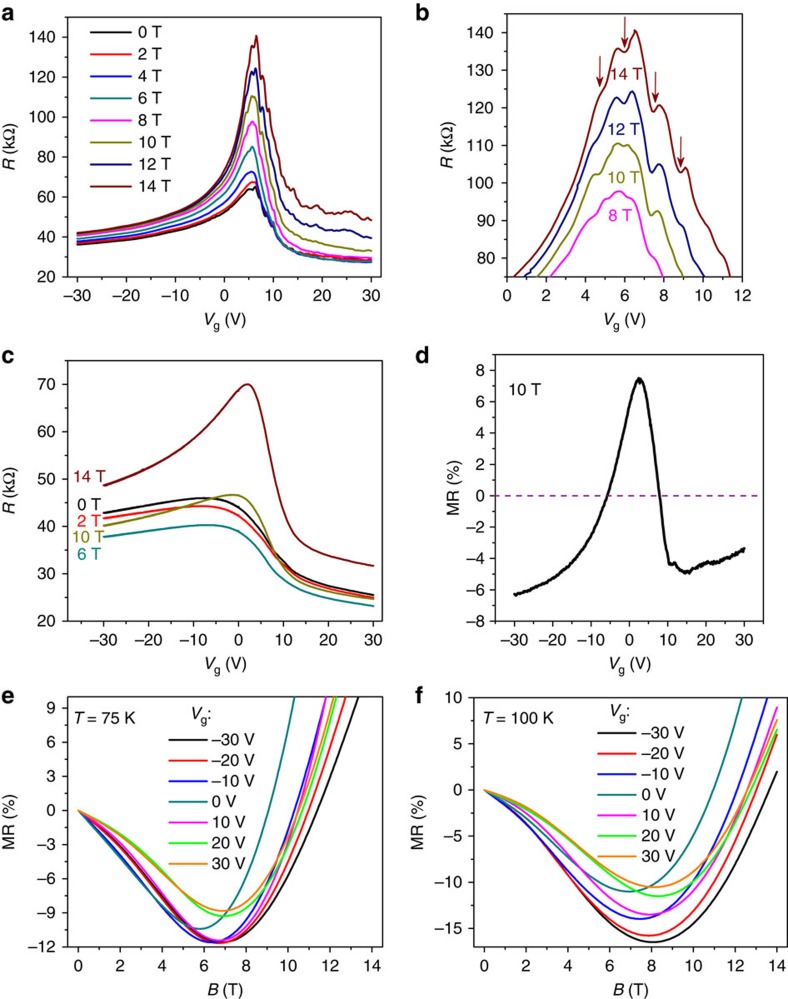
Gate-tunable transport properties with *B*//*E* of a nanowire with diameter ∼100 nm. (**a**) Transfer curves under different magnetic fields at 5 K and (**b**) the magnifications near the Dirac points for *B*≥8 T for Sample 2. Notable and repeatable peaks and valleys in the transfer curves are observed near the Dirac point under strong magnetic field due to splitting of the lowest Landau level. The resistance valleys are indicated by arrows. (**c**) Transfer curves under different magnetic fields at 75 K. No peaks and valleys in resistance are observed due to the thermal broadening of Landau level. (**d**) The MR(10 T)=[*R*(10 T)−*R*(0 T)]/*R*(0 T), obtained from the transfer curves under 0 and 10 T at 75 K, manifests a positive to negative transition as the gate voltage is tuned. (**e**,**f**) The MR curves measured at different gate voltages and at temperatures of 75 and 100 K, respectively.

To further reveal the Landau level splitting, we measured the gate voltage dependence of resistance of a nanowire with diameter ∼100 nm (Sample 2) under different magnetic fields as the Fermi level is tuned across the Dirac point, as shown in Fig. 3. By applying a strong magnetic field along the nanowire (*B*>8 T, *B*//*E*), notable and repeatable peaks and valleys in resistance are observed around the Dirac point whilst sweeping the gate voltage (Fig. 3a,b). Due to the low carrier density around the Dirac point in the Cd_3_As_2_ nanowire, the carriers are concentrated in the lowest Landau level under a relatively low magnetic field. Under high magnetic field, such as 14 T, the lowest Landau level splits into four sub-bands in energy scale, two in the conduction band, and the other two in the valence band (Supplementary Fig. 9). At *B*=14 T, the splitting of the zeroth Landau level is around 29 meV (ref. 18). As shown in Fig. 3b, the splitting of zeroth Landau level at 14 T is ∼1.5 V in Δ*V*_g_. Therefore, the position of the Fermi level with Dirac point located at 6.5 V in *V*_g_ can be estimated to be 47 meV according to *E*_F_∝*n*^1/3^∝Δ*V*_g_^1/3^, which is far less than 200 meV where Lifshitz transition occurs. As the Fermi level of the nanowire is tuned by sweeping gate voltage, the splitting of the lowest Landau level results in the resistance oscillations of the transfer curves. With the decrease of applied magnetic field, the resistance oscillations become weak and ultimately not distinguishable below 8 T. Because the maximum magnitude in negative MR occurred at a moderate temperature, we investigated the transfer curves under different magnetic fields at 75 K, as shown in Fig. 3c. By comparing the transfer curves at 0 and 10 T, a positive to negative MR transition is found whilst tuning the Fermi level away from the Dirac point, as shown in Fig. 3d. Under high magnetic field, as the Fermi level is close to the Dirac point, the energy gap breaks the chiral anomaly mechanism, resulting in the attenuation of the negative MR. The MR curves at *T*=75 K and 100 K and at different gate voltages are shown in Fig. 3e,f, which show the negative MR under low magnetic field and then a transition to positive MR at high magnetic field. It is clear that the magnitude of the negative MR and the *B*_C_ are tunable by gate voltage.

## Discussion

As the nanowires were synthesized by chemical vapour deposition method, different samples may have different defect concentration and thus different carrier density. The difference of the negative MR behaviours between different samples can be explained by considering the different carrier density. At *V*_g_=0 V and temperature around 75 K, the nanowire device with diameter ∼100 nm (Sample 2) has a critical magnetic field of ∼6 T (Fig. 3e) with MR magnitude of −10.5%, and nanowire device with diameter ∼200 nm (Sample 1) has a critical magnetic field of ∼7.5 T with MR magnitude of −55% (Fig. 2c). In other words, at *B*=10 T and *T*=75 K, the sample with low carrier density has entered into a positive MR-dominated region, while the sample with higher carrier density still shows negative MR and needs higher magnetic field (∼13 T) for positive MR arising.

**Figure 4:**
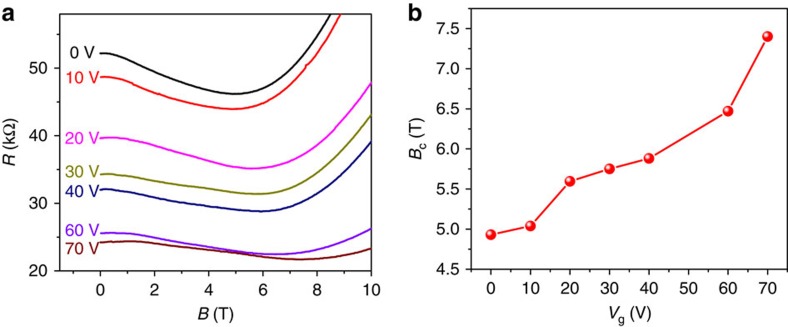
Gate-tunable-negative MR. (**a**) Resistance as a function of magnetic field at 60 K and at different gate voltages. The gate voltage is applied up to 70 V to enhance the carrier density largely. The magnitude of negative MR reduces with increasing gate voltage. (**b**) The critical magnetic field *B*_C_ as a function of gate voltage *V*_g_. The modulation of Fermi level away from the Dirac point gives rise to the increase of *B*_C_.

The shift of Fermi level with gate voltage and temperature can tune the carrier density. As shown in Fig. 3e,f, increase of carrier density by applying gate voltage results in the shift of the critical point of the MR curve towards higher magnetic field. Likewise, when increasing temperature from 75 to 100 K, thermal excitation gives rise to an increase of carrier density and the negative MR remains at *B*=10 T, as shown in Fig. 3f at *V*_g_=0 V. At room temperature, the high carrier density makes the Fermi level be far away from the Dirac point, and the negative MR was always observed in the *V*_g_ range from −30 to 30 V (Supplementary Fig. 10). The behaviours of the gate-modulated negative MR are repeatable, and the MR curves from another similar device measured at 60 K and at different gate voltages are shown in Fig. 4. To further lift the Fermi level into deeper conduction band, the applied gate voltage was increased up to 70 V. It is found that the magnitude of the negative MR decreases with the increase of gate voltage (Fig. 4a). Thus the absence of negative MR in bulk materials with high carrier density is not surprising^14^. Besides, the *B*_C_ increases with increasing gate voltage (Fig. 4b). As the gate voltage is increased, the Fermi level is tuned away from the Dirac point, giving rise to a larger critical point *B*_C_.

The finding of the lowest Landau level further indicates the achievable quantum limit configuration in our nanowire with low carrier density, which may be responsible for the absence of the Shubnikov-de-Haas (SdH) oscillations in the MR curves. Applying a low magnetic field, several Landau levels may be formed before reaching to the quantum limit, however the spacing between Landau levels is very small and can be submerged by disorder scattering and thermal broadening. Therefore, it is difficult to observe the SdH oscillations at low magnetic field in the Dirac system with low carrier density, which is consistent with the absence of SdH oscillations in the Dirac materials of Na_3_Bi (ref. 26) and graphene when the Fermi level is located close to the Dirac point^32^.

As the system enters into the extreme quantum limit at relatively low magnetic field due to the existence of a very small carrier concentration and high carrier mobility, the suppression of the back scattering can also result in a negative MR. Existing measurements in the literature have addressed the chiral anomaly effect in 3D Weyl and Dirac semimetals, which effectively illustrate the validity and reliability of the results^33^,^34^. Besides the negative MR in Cd_3_As_2_ nanowires, nonlocal measurements also support the chiral anomaly in Cd_3_As_2_, which may be the clear evidence to distinguish the suppression of the back-scattering effect and chiral anomaly effect^33^.

In summary, we have studied the magnetotransport properties of individual Dirac semimetal Cd_3_As_2_ nanowires. Benefiting from the single crystalline nature and the low carrier concentration of the nanowire, giant negative MR of −63% at ∼60 K is observed as the magnetic field is parallel to electric field. The negative MR is still notable with the value of −11% at 300 K. The observations give clear evidence for the chiral anomaly effect in the Cd_3_As_2_ system, which provides an ideal platform to explore new physical phenomena of 3D Dirac and Weyl semimetals.

## Methods

### Growth and characterization of the nanowires

The Cd_3_As_2_ nanowires were prepared by chemical vapour deposition method in tube furnace. Cd_3_As_2_ powders were placed at the centre of the furnace and silicon wafers coated with a thin gold film about 5 nm in thickness were used as substrates to collect the products downstream. First the tube furnace was flushed several times with Argon gas to fully remove oxygen. Then the temperature was gradually increased to 650 °C and kept for 10 min with an Argon flow of 20 s.c.c.m. as carrier gas. After the growth process, the furnace was cooled naturally. The SEM characterizations were performed in a FEI Nano430 SEM system, and the TEM characterizations were performed in a FEI Tecnai F20 TEM equipped with energy-dispersive X-ray spectroscopy system.

### Transport measurements

The synthesized Cd_3_As_2_ nanowires were transferred to a silicon substrate with an oxide layer of 285 nm, and then electrodes were fabricated after a series of processes including electron beam lithography, deposition of metal electrodes and lift-off. All the magnetotransport measurements were carried out in a commercial Oxford system, which can offer magnetic field up to 14 T and base temperature down to 1.5 K. Four-probe electrical measurements were performed through Stanford SR830 lock-in amplifiers by supplying 0.1 μA current with frequency of 17.7 Hz.

## Additional information

**How to cite this article:** Li, C.-Z. *et al.* Giant negative magnetoresistance induced by the chiral anomaly in individual Cd_3_As_2_ nanowires. *Nat. Commun.* 6:10137 doi: 10.1038/ncomms10137 (2015).

## Supplementary Material

Supplementary Information Supplementary Figures 1-10.
