# Supplementary material for: Giant negative magnetoresistance induced by the chiral anomaly in individual Cd3As2 nanowires
Source: Nat Commun. 2015 Dec 17;6:10137. doi: 10.1038/ncomms10137 (PMC4703844; doi:10.1038/ncomms10137)
Supplement: Supplementary Information — Supplementary Figures 1-10. [file ncomms10137-s1.pdf]

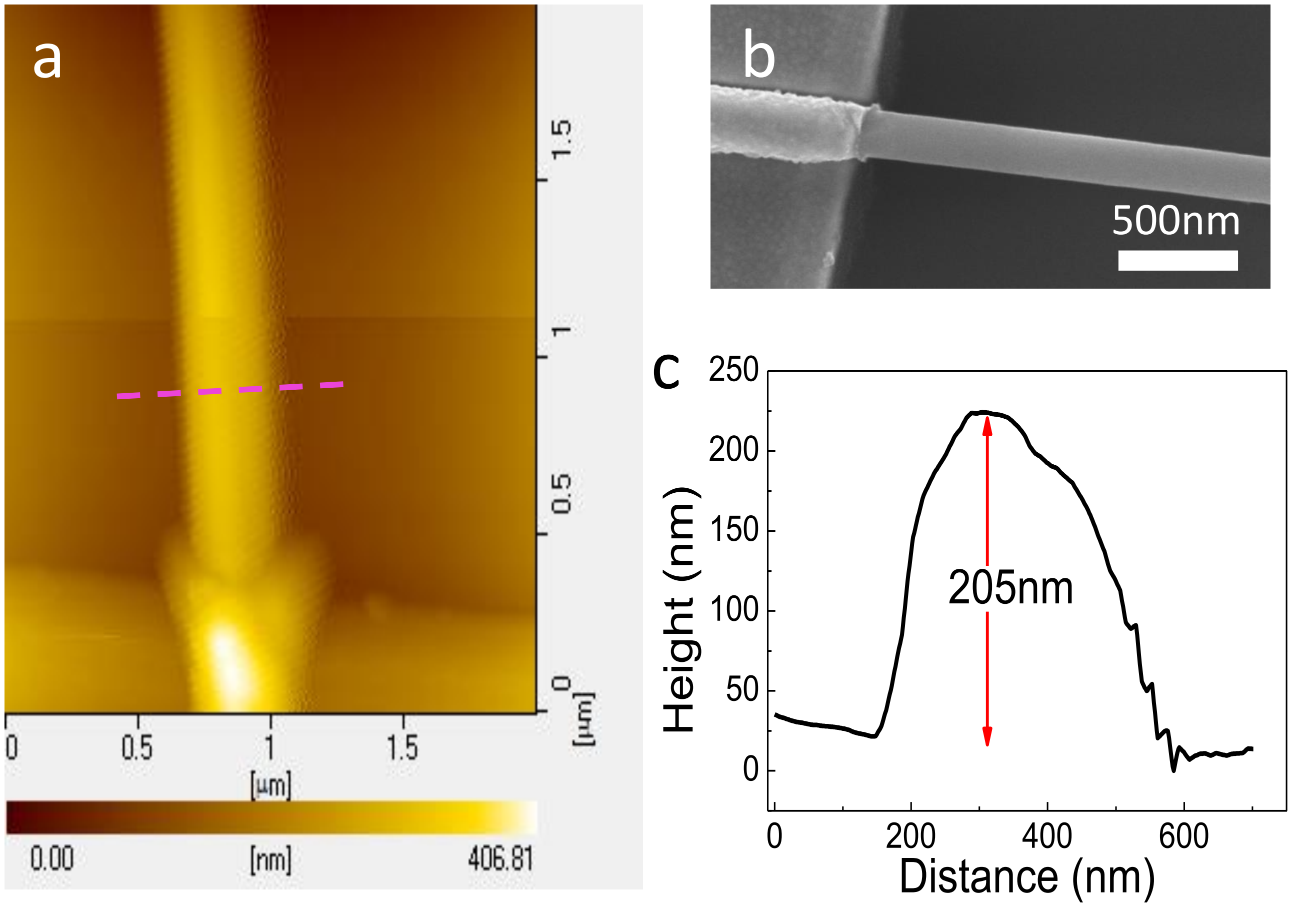

Supplementary Figure 1. Geometric parameters. (a) The atomic force microscope (AFM) image of a typical device with diameter of ~ 200 nm. (b) The scanning electron microscope (SEM) image of the device. (c) The height profile of this nanowire along the pink line (marked in a).

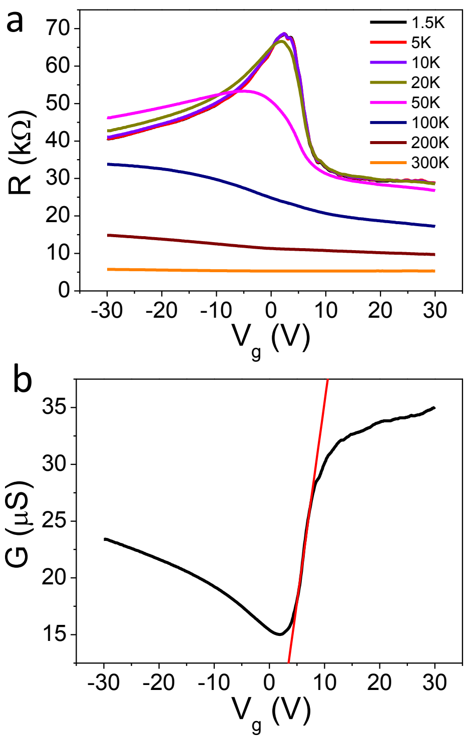

Supplementary Figure 2. Calculations of the carrier concentration. (a) Transfer curves of a typical nanowire device with diameter of ~100 nm at different temperatures from 1.5 K to 300 K. The Dirac point V_D locates near $V_g = 0V$ at low temperatures, indicating the low carrier density. With increasing temperature, the V_D shifts towards negative gate voltage. At temperatures above 100 K, the V_D cannot be observed in the range of V_g from -30 to 30 V, because more carriers are thermally activated. The carrier concentration is obtained by $n = \frac{C}{l}\frac{1}{eS}\left(V_g - V_D\right)$, where $\frac{C}{l} = \frac{\pi\varepsilon_0\varepsilon_r}{cosh^{-1}(\frac{R+h}{R})}$,[1] C is the capacitance of the oxide layer (SiO_2), $\varepsilon_r = 3.9$ is the relative dielectric constant of SiO_2, h = 285 nm is the SiO_2 thickness, R, l, and S are the radius, length, and cross-section area of the nanowire, respectively. The carrier mobility can be calculated by $\mu = \frac{\partial G}{\partial V_g} \times \frac{l^2}{C}$, where G is the conductance. (b) The fitting of the conductance (G) *vs.* gate-voltage (V_g) dependence at 20 K. The red line represents the linear fit. The carrier mobility fitted from the transfer curve at 20 K is about 1.68×10^3 $cm^2V^{-1}s^{-1}$. At $V_g = 0\,V$, the calculated carrier concentrations n are 0.8×10^{17}, 0.5×10^{17}, and -1.3×10^{17} cm^{-3} at temperature of 5 K, 20 K, and 50 K, respectively. Here the $n > 0$ corresponds to the hole dominant transport, while $n < 0$ corresponds to the electron dominant transport.

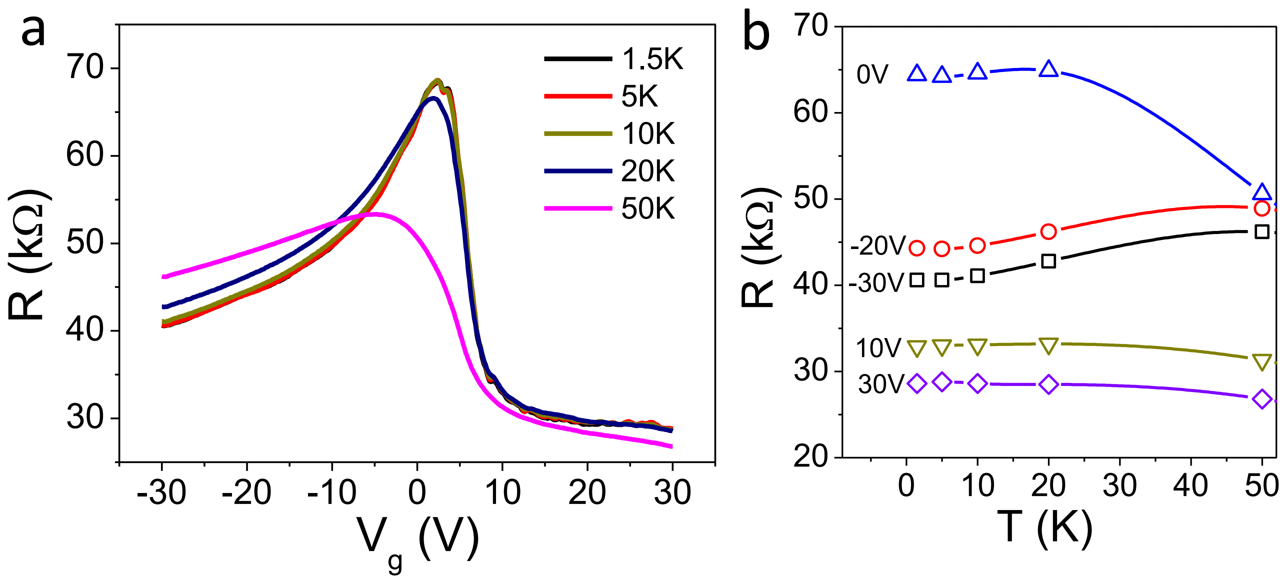

Supplementary Figure 3. Gate-tunable R-T dependence. (a) Transfer curves of a typical nanowire device with diameter of ~100 nm at different temperatures from 1.5 K to 50 K. It is the magnification of the Supplementary Figure 2a. The Dirac point (V_D) of the transfer curve shifts toward the negative gate voltage (V_g) as increasing temperature, which is consistent with the physical picture of thermal activation of the electrons in the valence band to the conduction band. (b) Temperature dependence of resistance at different V_g. At V_g = 0 V there is a semiconducting-like R-T behaviour. At V_g = 30 V, the Fermi level is much above the Dirac point and much less electrons in the valence band can be thermally activated to the conduction band, resulting in a very weak temperature dependence of resistance. At V_g = -30 V, the Fermi level is in the valence band, and there is a metallic R-T behavior as the electrons below the Fermi level cannot be thermally activated. It is worth to note that the R-T behavior may be slightly different from sample to sample at low temperatures due to the different residual carrier density in different samples. For the sample with very low residual carrier density, the semiconducting-like R-T relationship will extend to low temperature. While for the sample with high residual carrier density, there is a transition from the semiconducting-like R-T relationship at relatively high temperature to the metallic R-T behavior at low temperatures.

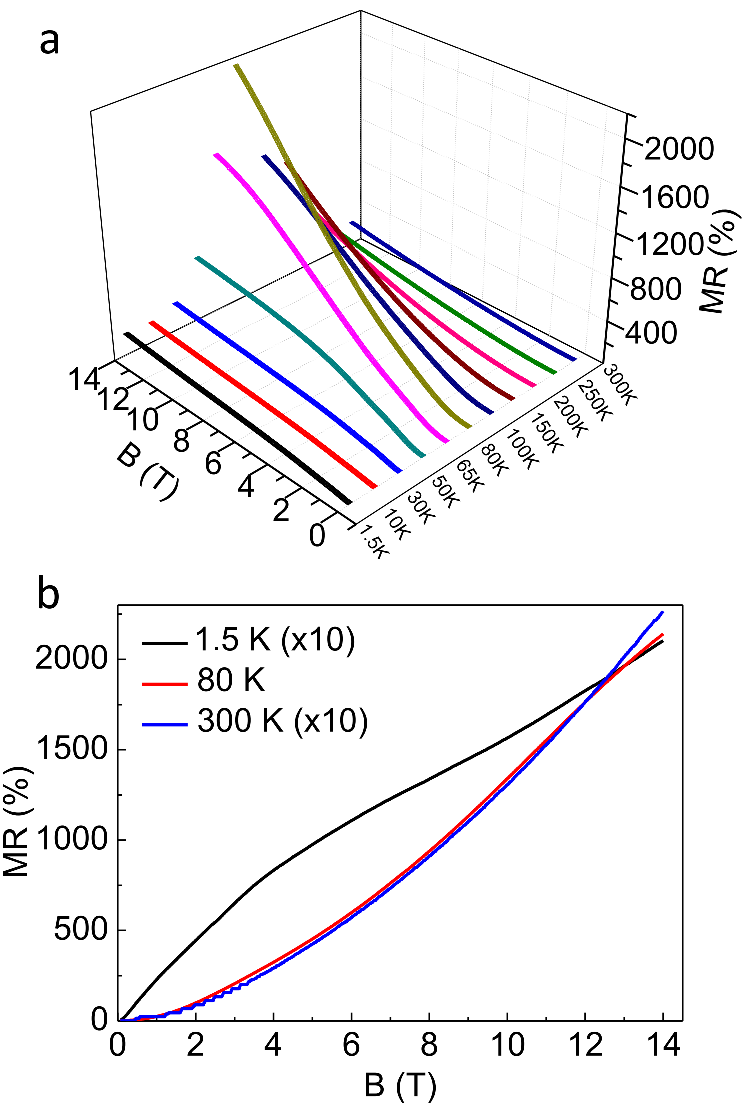

Supplementary Figure 4. Giant positive MR under perpendicular fields. (a) The MR behaviors of a nanowire device with diameter about 230 nm at different temperatures. Under B=14 T, the MR is 210% at 1.5 K, and reaches the maximum of 2140% at 80 K, then reduces to 226% at 300 K. The largest MR appeared at moderate temperature range is in accordance with the non-metallic $\rho - T$ behavior. On one hand, at high temperatures (T > 100 K), the Landau level at high magnetic field is thermally smeared and the magnetic field induced resistance change is relatively small compared with that at low temperatures. On the other hand, at low temperatures (T < 30 K), the zero-field resistance R (B=0) is very large. Since the MR = R(14 T)/R(0 T) -1, it is expected to observe the largest MR at moderate temperatures. (b) The MR at 1.5 K and 300 K are magnified 10 times and plotted with the MR at 80 K for comparison. In the low magnetic field region, the MR at 1.5 K increases fast and can be attributed to the weak anti-localization effect, while the MR at 300 K shows a quadratic increase and is due to the classical theory of the carriers with Lorentz force.

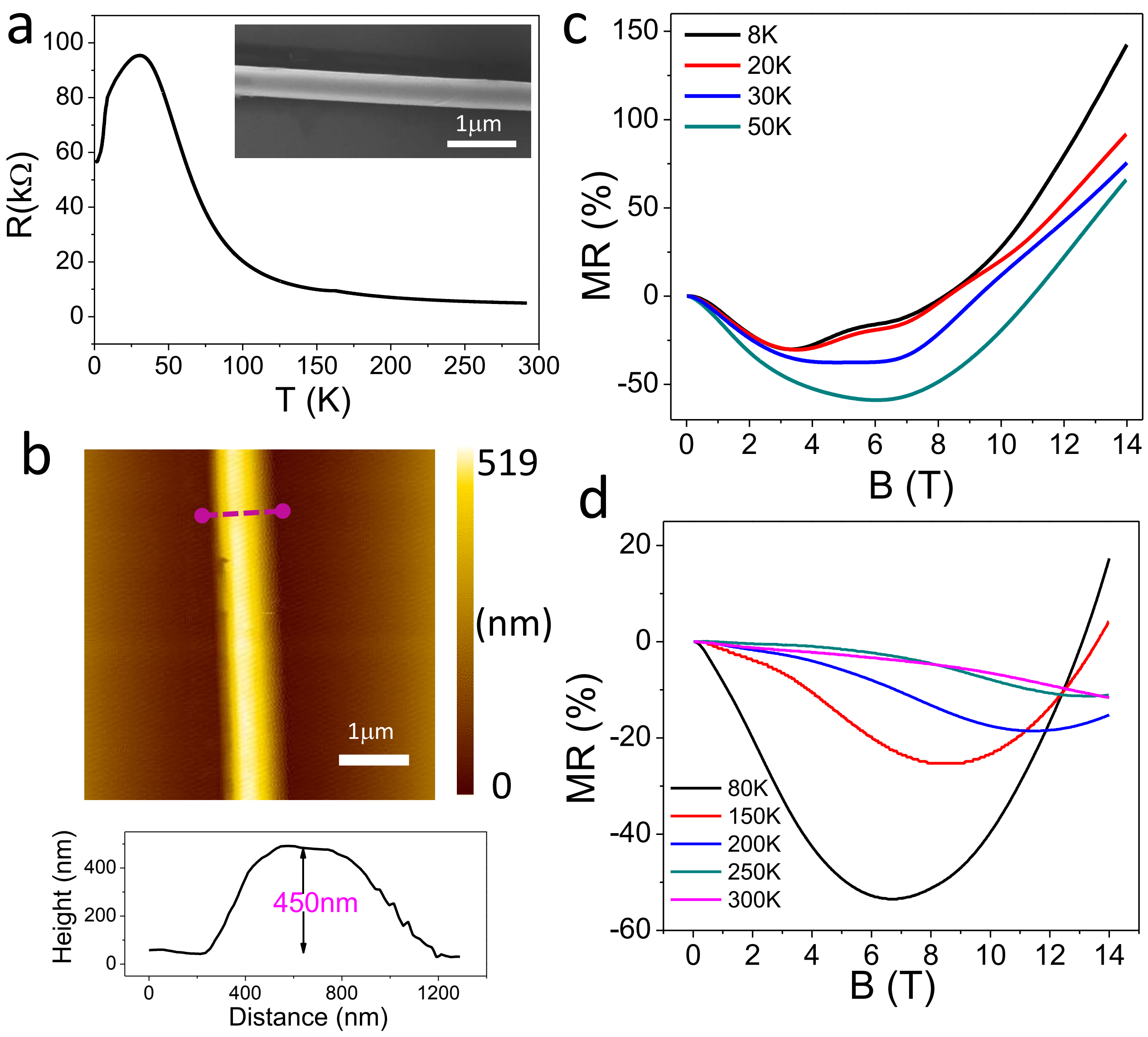

Supplementary Figure 5. Negative MR of a nanowire with diameter ~450 nm. (a) Temperature dependence of resistance of the device and its SEM image (inset). (b) AFM results of the nanowire device with height about 450 nm. (c)-(d) Plots of MR =100% × (R(B)/R(0)-1) at variable temperatures. The magnitude of the negative MR reaches maximum ~-59% at a moderate temperature of 50 K.

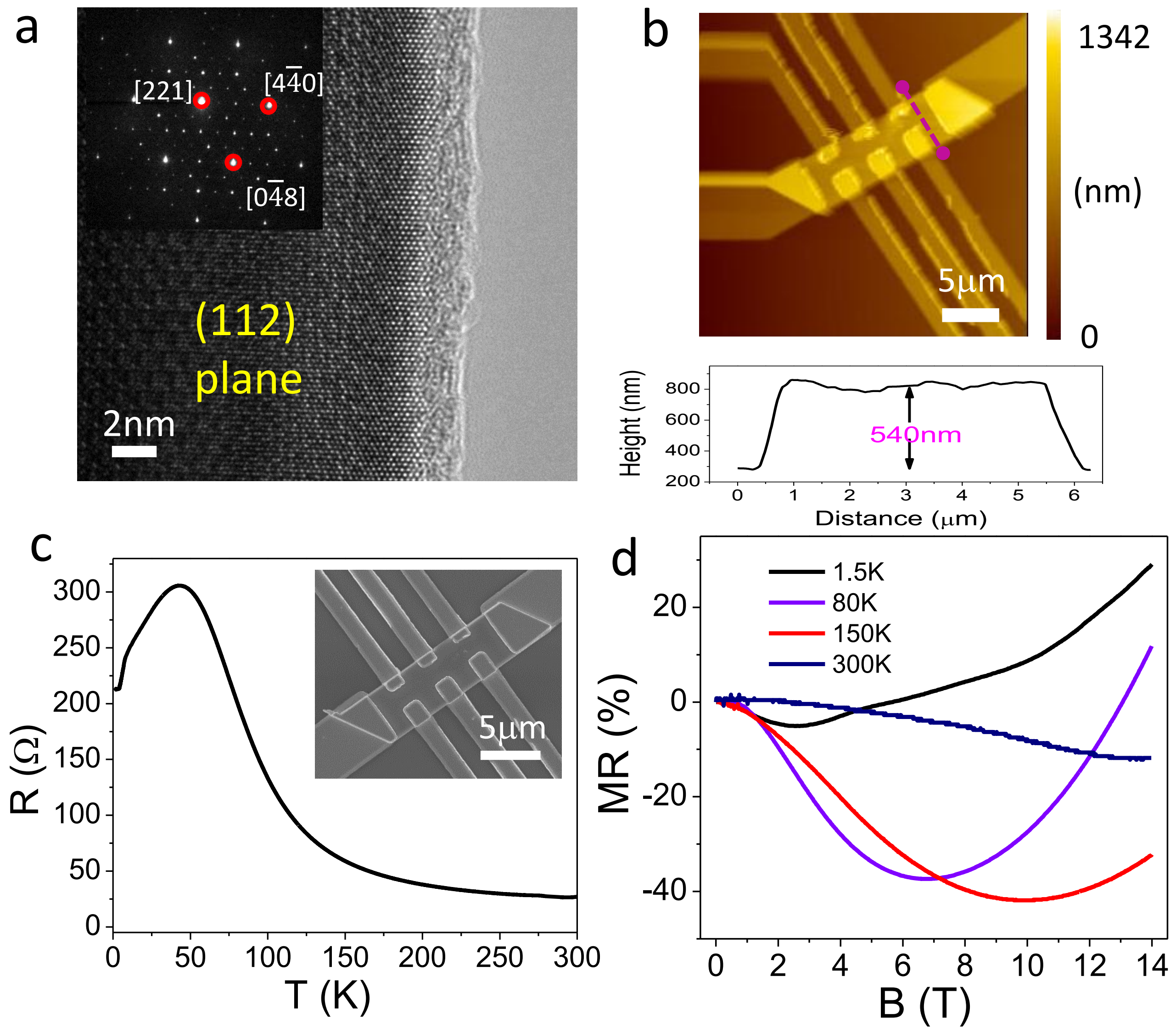

Supplementary Figure 6. Negative MR of a nanoplate with thickness ~540 nm. (a) TEM image of a typical nanoplate that identifies its largest surface to be a (112) plane. Inset: the corresponding diffraction pattern. (b) AFM measurement of the nanoplate device with height about 540 nm. (c) Temperature dependence of resistance of the nanoplate device and its SEM image (inset). (d) Plots of MR =100%×(R(B)/R(0)-1) at variable temperatures. The magnitude of the negative MR reaches maximum ~-41% at ~ 150 K.

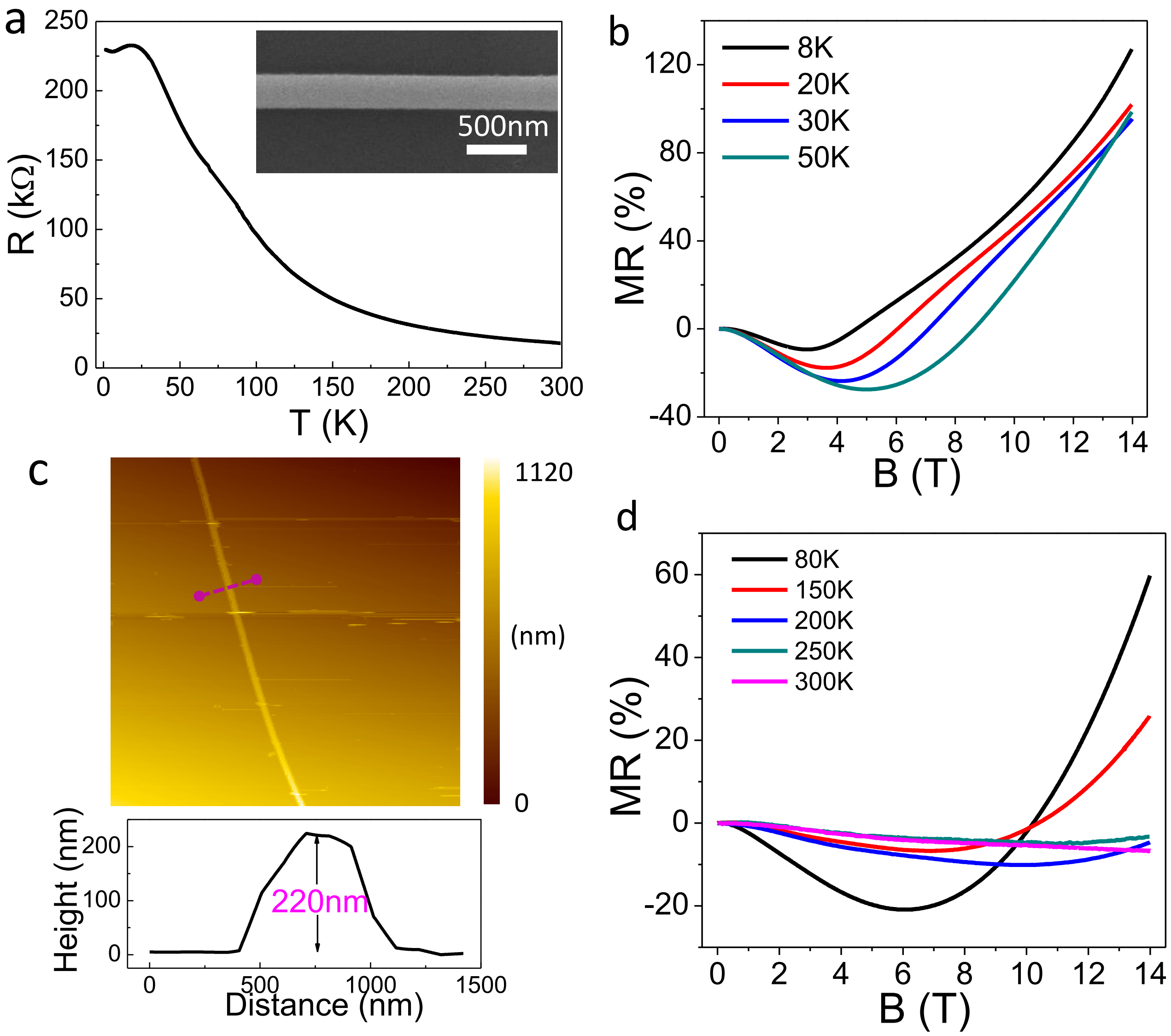

Supplementary Figure 7. The device with relatively small negative MR. (a) Temperature dependent resistance of a nanowire device and its SEM image (inset). The device still shows non-metallic R-T behavior at low temperatures, indicating the low residual carrier density. (b) AFM results of the nanowire device with height about 220 nm. (c)-(d) Plots of MR =100%×(R(B)/R(0)-1) at different temperatures. The negative MR is relatively small compared with the devices with metallic R-T behavior at low temperatures, such as the data presented in Figure 2.

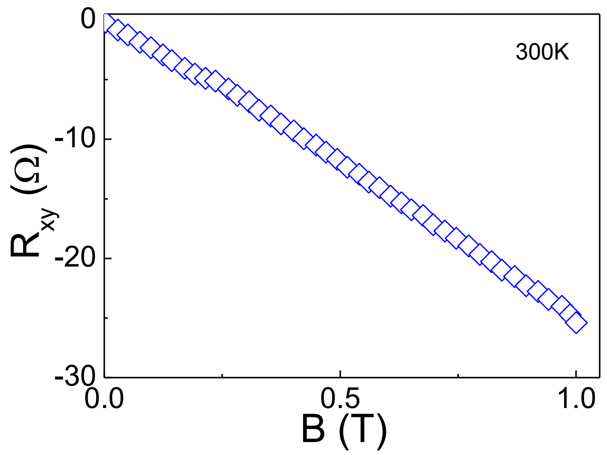

Supplementary Figure 8. Hall resistance. The Hall resistance of a nanoplate with thickness of 540 nm at 300 K and under low magnetic field. The Cd_3As_2 nanoplate still has low carrier density even with considerable thermal activation at 300 K. The carrier density is estimated to be $5.31 \times 10^{17} cm^{-3}$, lower than that in bulk crystals, which is critical for the observation of negative MR.

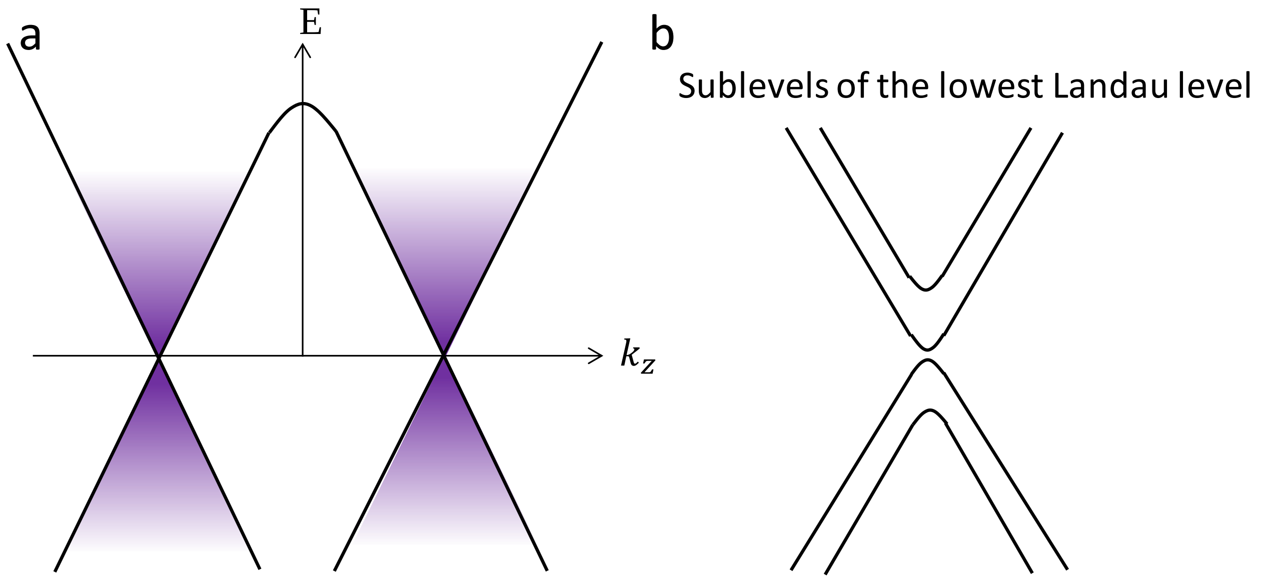

Supplementary Figure 9. Splitting of the lowest Landau level. (a) The band structure of Cd_3As_2 with two Dirac points. (b) Splitting of the lowest Landau levels under magnetic field in [112] direction at one Dirac point. The Dirac point is constituted by a pair of spin-degenerate Weyl nodes. Under high magnetic field, such as 14 T, the lowest LL splits into four sub-bands in energy scale, two in the conduction band, and the other two in the valence band. As tuning the Fermi-level passes through each of the sublevels by gate voltage, a valley in resistance (or a peak in conductance) appears.

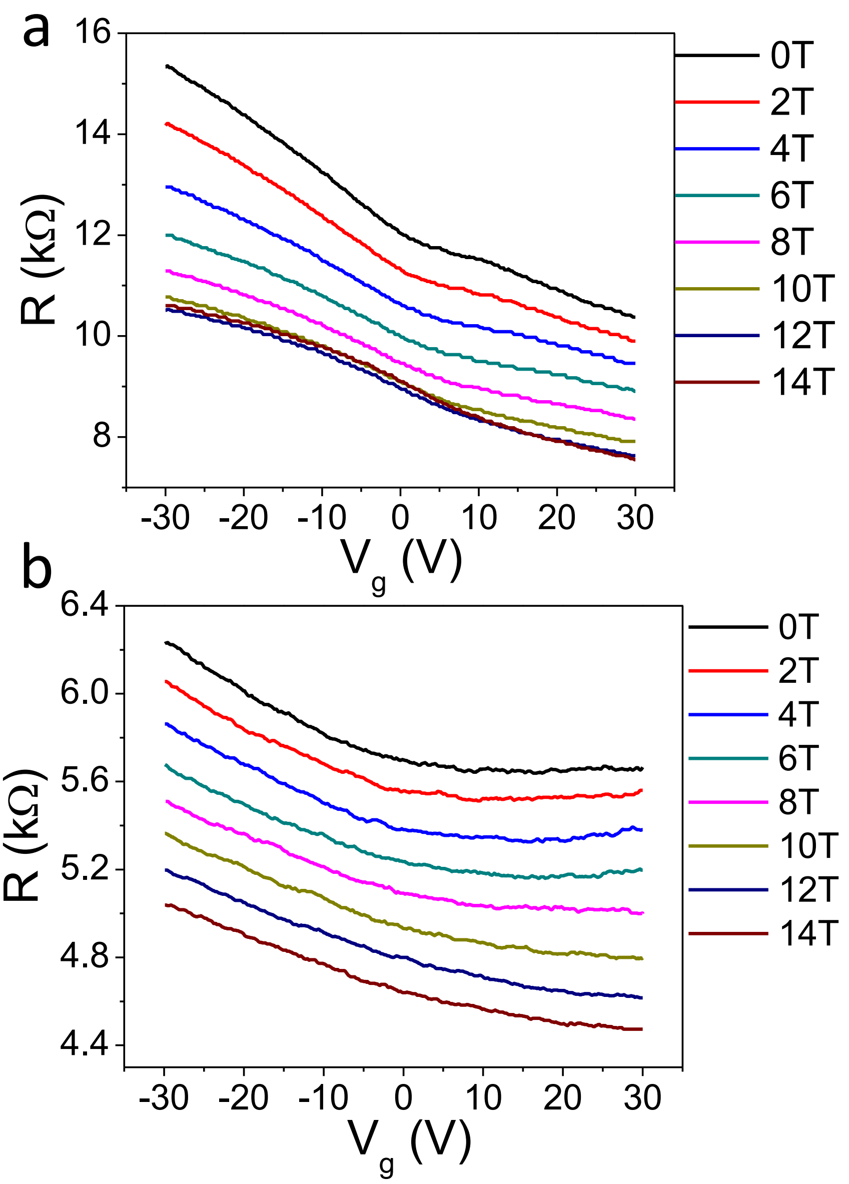

Supplementary Figure 10. Negative MR at high temperatures. Transfer curves of a nanowire with diameter ~ 100 nm (Sample 2) under different magnetic fields at (a) 200 K and (b) 300 K. The negative MR is clearly observed at 200 K and 300 K. At high temperatures, the carrier density is high and the transport is dominated by electrons. The Dirac point can not be reached in the V_g range -30 ~ 30 V. The negative MR is still observed even at 14 T.
